# Identification of immune‐related hub genes and construction of an immune‐related gene prognostic index for low‐grade glioma

**DOI:** 10.1111/jcmm.17960

**Published:** 2023-09-29

**Authors:** Jing Zhou, Hao Guo, Likun Liu, Zengcai Jin, Wencui Zhang, Tao Tang

**Affiliations:** ^1^ Department of Oncology, Shanxi Province Academy of Traditional Chinese Medicine Shanxi Province Hospital of Traditional Chinese Medicine Taiyuan China; ^2^ Department of Anesthesiology Shanxi Provincial People's Hospital Taiyuan China; ^3^ Department of Integrated Traditional Chinese and Western Medicine, Institute of Integrative Medicine Xiangya Hospital, Central South University Changsha China; ^4^ National Clinical Research Center for Geriatric Disorders Xiangya Hospital, Central South University Changsha China

**Keywords:** immune checkpoint inhibitors, immunotherapy, low‐grade glioma, tumour immune microenvironment, WGCNA

## Abstract

Low‐grade glioma (LGG) poses significant management challenges and has a dismal prognosis. While immunotherapy has shown significant promise in cancer treatment, its progress in glioma has confronted with challenges. In our study, we aimed to develop an immune‐related gene prognostic index (IRGPI) which could be used to evaluate the response and efficacy of LGG patients with immunotherapy. We included a total of 529 LGG samples from TCGA database and 1152 normal brain tissue samples from the GTEx database. Immune‐related differentially expressed genes (DEGs) were screened. Then, we used weighted gene co‐expression network analysis (WGCNA) to identify immune‐related hub genes in LGG patients and performed Cox regression analysis to construct an IRGPI. The median IRGPI was used as the cut‐off value to categorize LGG patients into IRGPI‐high and low subgroups, and the molecular and immune mechanism in IRGPI‐defined subgroups were analysed. Finally, we explored the relationship between IRGPI‐defined subgroups and immunotherapy related indicators in patients after immunotherapy. Three genes (RHOA, NFKBIA and CCL3) were selected to construct the IRGPI. In a survival analysis using TCGA cohort as a training set, patients in the IRGPI‐low subgroup had a better OS than those in IRGPI‐high subgroup, consistent with the results in CGGA cohort. The comprehensive results showed that IRGPI‐low subgroup had a more abundant activated immune cell population and lower TIDE score, higher MSI, higher TMB score, lower T cell dysfunction score, more likely benefit from ICIs therapy. IRGPI is a promising biomarker in the field of LGG ICIs therapy to distinguish the prognosis, the molecular and immunological characteristics of patients.

## 1 INTRODUCTION

Glioma is the most common primary central nervous system (CNS) malignant tumour with a very poor prognosis.^1^, ^2^, ^3^, ^4^, ^5^ Despite the use of surgical resection, radiotherapy and systemic drug chemotherapy in low‐grade glioma (LGG) patients, the tumour recurrence and malignant progression was still common.^6^, ^7^ The results in a significant difference in the prognosis of patients.

Immunotherapy has shown remarkable progress in the field of tumour treatment, especially PD‐1/PD‐L1 and CTLA‐4 immune checkpoint inhibitors (ICIs), which have significantly improved the prognosis of patients.^8^, ^9^ However, the progress of immunotherapy in the field of glioma has confronted with challenges.^10^ The blood brain barrier (BBB) can selectively block the transmission of immune cells or immune macromolecules from peripheral to CNS.^11^ Additionally, the unique immune environment in the CNS is nonnegligible. The tumour microenvironment (TME) of glioma comprises resident microglia and surrounding immune cells.^12^ Tumour‐derived cytokines and chemokines are capable of reprogramming these immune cells into tumour‐associated phenotypes, ultimately leading to malignant progression or drug resistance.^13^ Moreover, the T cells were less infiltrated in the immune landscape of glioma.^14^, ^15^ A variety of factors including TME can affect the immunotherapy, but few reliable immune markers can accurately predict the prognosis of patients clinically. Therefore, to address these challenges, it is essential to explore new tumour intervention targets and develop more effective clinical prediction models for accurately evaluating the prognosis of LGG.

The purpose of this study was to develop a novel clinical prognostic index to evaluate the response and effectiveness of immunotherapy in LGG patients. Initially, we collected the RNA sequencing data of LGG samples and their clinicopathological information, as well as normal brain tissue samples for comparative analysis. Immune‐related differentially expressed genes (DEGs) were screened. Then, we identified the immune‐related hub genes related to patient prognosis using weighted gene co‐expression network analysis (WGCNA). Furthermore, an immune‐related gene prognostic index (IRGPI) was determined and constructed by regression analysis. Finally, we explored the molecular and immunological characteristics of IRGPI and verified its prognostic ability in patients with LGG and other cancer types. The results of comparison with tumour immune dysfunction and exclusion (TIDE) and tumour inflammation signature (TIS) further showed that IRGPI could be a promising biomarker in the field of LGG therapy.

## 2 MATERIALS AND METHODS

**FIGURE 1:**
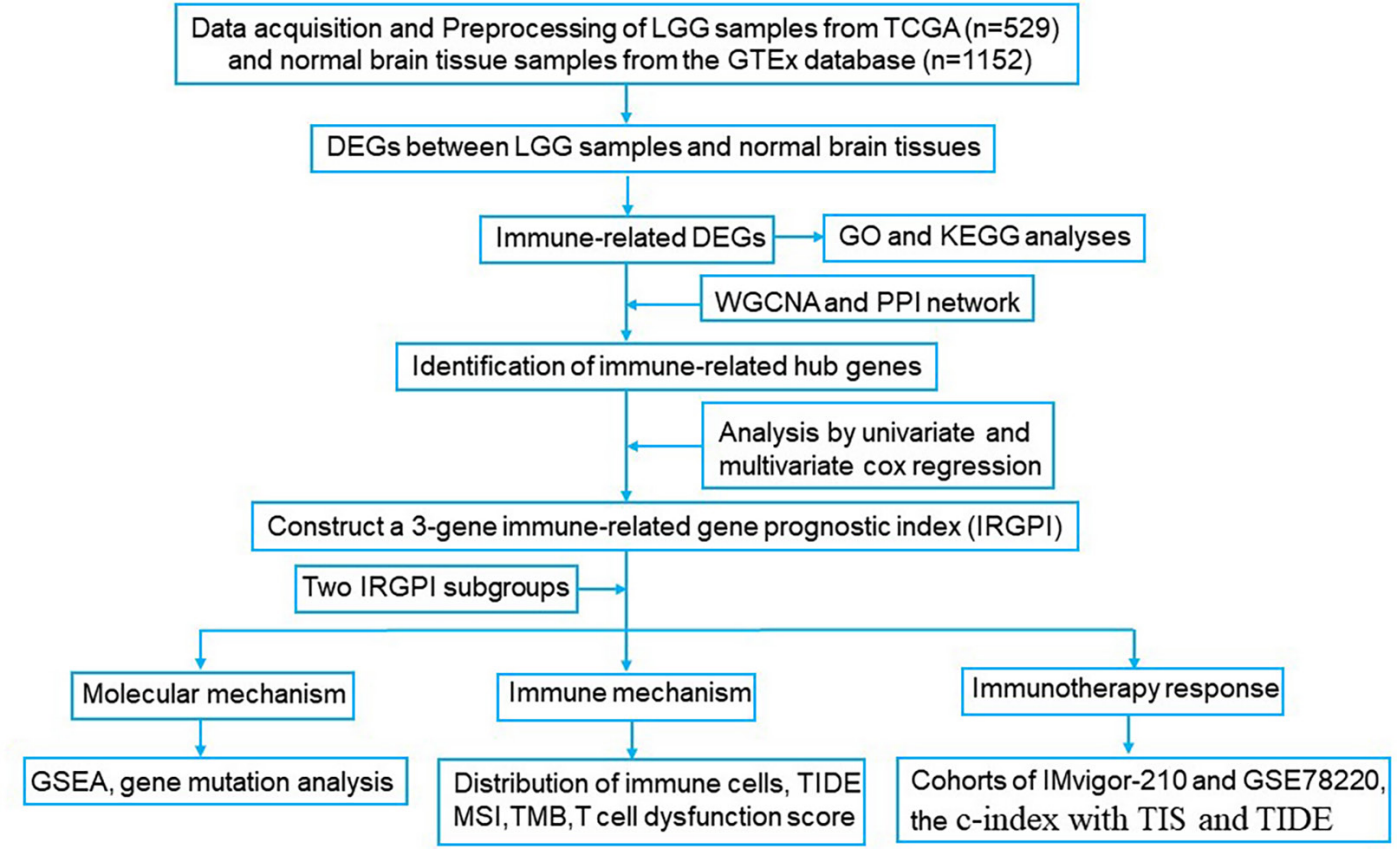
The workflow chart of this study design and analysis.

The workflow of this study is shown in Figure 1.

### 2.1 Data acquisition and preprocessing

RNA‐sequencing data and all clinicopathological information of 529 LGG samples were obtained from the Cancer Genome Atlas database (TCGA, https://www.cancer.gov/tcga) database. 1152 normal brain tissue samples were downloaded from the GTEx database (https://commonfund.nih.gov/gtex) for differential analysis. The RNA‐seq data and survival information of LGG samples were downloaded from the Chinese Glioma Genome Atlas database (CGGA, http://cgga.org.cn/index.jsp).

The list of immune‐related genes was downloaded from the ImmPort (https://www.immport.org/shared/home) database. The Networks of relationship among RNAs, including transcription factors (TFs) and miRNA were obtained from NetworkAnalyst (https://dev.networkanalyst.ca/) database. The detailed information on gene mutation was obtained from the cBioPortal (http://www.cbioportal.org/) database. Regarding the experimental code, we have made the code publicly available on GitHub and included a link (https://github.com/sooc‐how‐Feng/LGG‐subtypes) to the repository.

### 2.2 WGCNA and identification of immune‐related hub genes

We used the ‘limma’ R package to identify DEGs between LGG samples and normal brain tissues (*p* < 0.05, | log2FC| > 1). Further, we selected immune‐related DEGs by integrating the immune‐related gene lists from ImmPort. Subsequently, Gene Ontology (GO) and Kyoto Encyclopedia of Genes and Genomes (KEGG) analyses were conducted on these genes using the ‘clusterProfiler’ R package.

Unlike the conventional approach of focusing solely on DEGs, we employed the WGCNA to identify gene sets that had a significant association with clinical phenotypes using all gene information. To construct the gene co‐expression network of immune‐related DEGs.^16^ We first generated the similarity matrix based on the Pearson correlation coefficient between every two genes. Next, we transformed the similarity matrix into an adjacency matrix by screening a suitable soft threshold. The topological overlap matrix (TOM) was then constructed to estimate the connectivity of genes in the network. We applied the dynamic shearing method to form modules with different traits based on the clustered genes. Finally, we identified four modules by setting the merging threshold function at 1.0. The identification of immune‐related hub genes was carried out by constructing PPI network and setting weight thresholds on the basis of module genes significantly related to tumour traits. The top 50 genes ranked by the degree of connectivity in the network were selected as the hub genes. Using the ‘maxstat’ R package, we obtained the optimal cut‐off value for the overall survival (OS) of each hub gene. To further improve the prognostic guidance, we selected immune‐related hub genes significantly associated with patient survival for subsequent analysis.

Somatic mutations of immune‐related hub genes were obtained using the ComplexHeatmap package of R to reveal the changes in genetic traits related to genes. The roles of these genes in TFs and miRNA regulatory networks were analysed, and KEGG pathway enrichment analysis was also performed to determine their potential regulatory mechanisms in tumours.

### 2.3 Construction and validation of the immune‐related gene prognostic index

We utilized regression analysis to select immune‐related hub genes for constructing the model. To reduce collinearity, we further employed LASSO regression analysis, followed by multivariate Cox regression analysis to identify the genes significantly affecting OS. These selected genes were used to construct an IRGPI. The IRGPI of each sample was the final sum of the product of the expression value of each gene and its weight in the model.

We evaluated the independence of prognostic value for IRGPI using univariate and multivariate Cox regression analysis. Then, we divided LGG patients into different subgroups based on their IRGPI scores calculated using the median IRGPI scores as the cut‐off value. We also performed Kaplan–Meier survival curves with log‐rank tests to validate the prognostic value of IRGPI in both TCGA and CGGA cohorts. Besides, our research integrated age, gender, IDH status, WHO grade and 1p/19q codeletion with IRGPI to further explain that IRGPI had advantages in predicting the prognosis of LGG patients compared with other clinical indicators.

### 2.4 Comprehensive analysis of immune landscape in different IRGPI subgroups

To comprehensively investigate the underlying cause of prognostic variation among different IRGPI subgroups, we employed various pathway analysis. First, the DEGs between different subgroups of IRGPI were identified using the ‘limma’ R package. Next, these genes that involved in the KEGG and HALLMARK gene sets were analysed by the gene set enrichment analysis (GSEA) to determine the gene sets and core pathways enriched in different IRGPI subgroups. We also conducted single‐sample GSEA (ssGSEA) analysis on the gene sets of interest and examined the survival differences through Kaplan–Meier survival curves.

To further elucidate the immune‐related characteristics of different subgroups of the IRGPI, we conducted gene mutation analysis using the ‘Maftools’ package of R in different IRGPI subgroups obtained from the cBioPortal database. Moreover, we used the CIBERSORT algorithm (https://cibersort.stanford.edu/) to calculate the relative proportions of 22 types of immune cells in the LGG samples. Finally, in combination with clinicopathological factors, we depicted the landscape of the proportions of immune cell infiltrations among different IRGPI subgroups.

### 2.5 The prognostic value of IRGPI in patients after immunotherapy

To assess the prognostic relevance of IRGPI in patients receiving immunotherapy, we conducted a survival analysis in the cohort of IMvigor‐210 (urothelial carcinoma, UC) treated with anti‐PD‐L1 therapy and GSE78220 (melanoma) treated with anti‐PD‐1 therapy.^17^ Furthermore, we compared the prognostic value of IRGPI with TIDE and TIS using the ‘timeROC’ package in R. We obtained the concordance index (C‐index) value by performing a time‐dependent ROC curve analysis.

### 2.6 Statistical analysis

Statistical analyses were conducted using SPSS version 22.0 and R version 3.6.1, along with their appropriate packages. An independent *t*‐test was used to compare continuous variables between the two groups, while the chi‐squared test was used for classification data. Regression analysis was used to select immune‐related hub genes and construct the model, while univariate Cox regression analysis was employed to identify genes closely associated with OS. Multivariate Cox regression analysis was conducted to perform multivariate analysis and construct IRGPI. The prognostic value of IRGPI was evaluated using receiver operating characteristic (ROC) curve analysis and the corresponding area under the ROC curve (AUC). All statistical tests were two‐sided, and a *p*‐value less than 0.05 was considered statistically significant.

## 3 RESULTS

### 3.1 Identification of immune‐related hub genes and comprehensive analysis of their potential mechanisms

**FIGURE 2:**
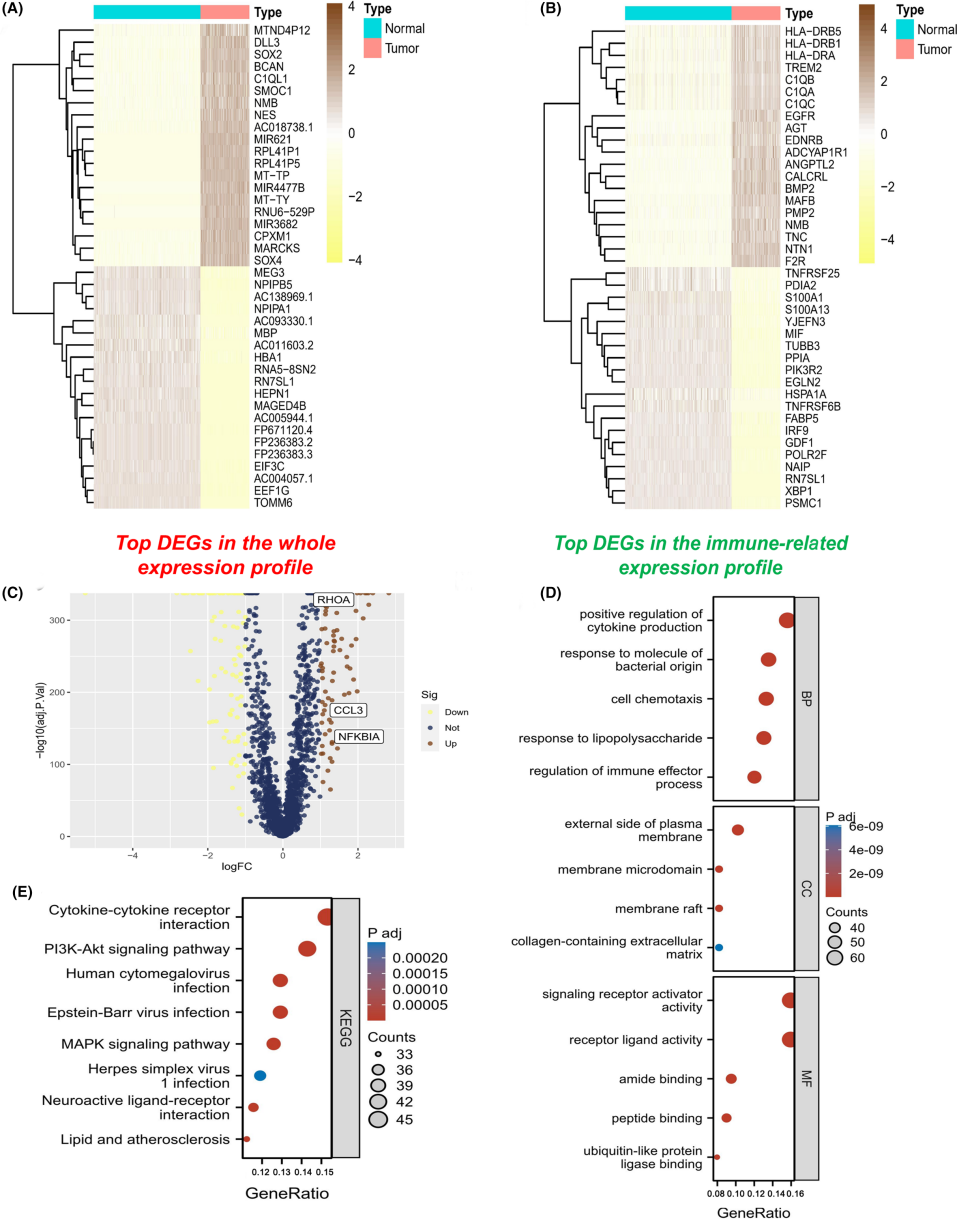
Differentially expressed immune‐related genes between LGG samples and normal brain tissues. (A) The top differentially expressed genes (DEGs) between LGG and normal samples. (B) The top immune‐related DEGs between LGG and normal samples. (C) The volcano plot for the immune‐related DEGs (*p* < 0.05, | log2FC| > 1). (D) Gene Ontology (GO) enrichment analysis and (E) Kyoto Encyclopedia of Genes and Genomes (KEGG) pathway analysis of the immune‐related DEGs.

To identify immune‐related DEGs, we first performed differential expression analysis on tumour samples and normal samples. Among them, 4401 DEGs were identified. Of these, 1849 genes were upregulated and 2552 genes were downregulated in tumour samples compared with normal samples (Figure 2A). We then intersected these DEGs with a list of immune‐related genes and identified 396 immune‐related DEGs, with 241 genes upregulated and 155 genes downregulated in tumour samples (Figure 2B,C). The functional enrichment analysis of these genes showed that 396 immune‐related DEGs were significantly correlated with 2317 GO terms and 149 KEGG pathways (Table S1), of which the top GO terms and KEGG pathways are shown in Figure 2D,E.

**FIGURE 3:**
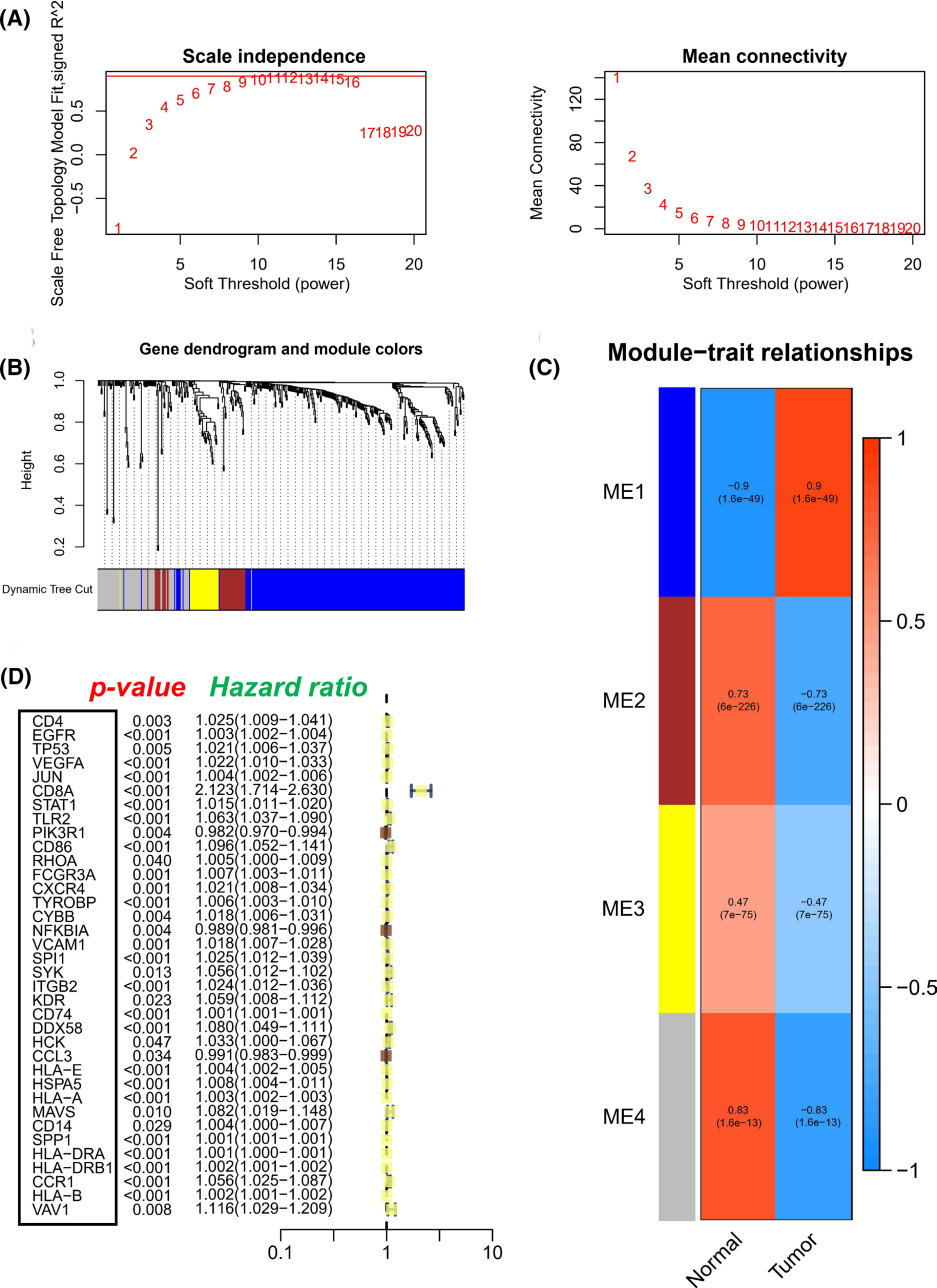
Weighted co‐expression network construction and key modules identification by WGCNA. (A) The scale‐free topological fit index and the average connectivity was calculated to determine the optimal soft threshold power (*β* = 10) of the WGCNA. (B) Clustering dendrograms and modules identified of immune‐related differentially expressed genes by WGCNA. (C) Heatmap of the correlation between module eigengenes and the disease of LGG. (D) The relationships between the expression profiles of 36 immune‐related hub genes and OS. In these 36 immune‐related DEGs, yellow squares display risk factors and the other three brown squares display protective factors.

To identify immune‐related hub genes for LGG patients, we constructed a co‐expression network for 396 candidate genes via the R package of WGCNA. We removed genes with zero variance between groups and used the hclust function to form clustering dendrograms. The soft threshold was set as 10 to ensure that the constructed network had the characteristics of a scale‐free network (Figure 3A), and we identified four modules according to average linkage hierarchical clustering (Figure 3B). By calculating the correlation coefficients between each module and clinical traits, we finally determined that ME1 and ME4 modules were closely related to tumours (Figure 3C). Subsequent analysis of genes in these modules allowed us to uncover underlying mechanisms of tumour development. The top GO terms and KEGG pathways with significant enrichment of genes in ME1 and ME4 modules are shown in Figure S1A,B (details in Table S2).

We constructed a PPI network with a threshold weight > 0.4 for the ME1 and ME4 modules, respectively and shown the core group in each module (Figure S1C,D). Based on network connectivity, we identified the top 50 genes as immune‐related hub genes. Univariate cox regression analysis of these 50 hub genes further identified 36 immune‐related hub genes closely related to OS in LGG patients (Figure 3D).

Next, we investigated the underlying mechanism of these 36 immune‐related hub genes in LGG patients. Our results indicated that these genes frequently underwent amplification, deep deletion and missense mutation. Notably, TP53, EGFR, CD4 and PIK3R1 had a mutation frequency of more than 5% (Figure S2A). Furthermore, analysis of the regulatory network of TFs and miRNA revealed that these 36 hub genes had a significant interaction with TFs and miRNA, with 369 pairs of interactions with TFs and 785 pairs of interactions with miRNA (Figure S2B). KEGG pathway enrichment analysis showed that these genes were mainly enriched in signalling pathways such as positive regulation of tumour necrosis factor superfamily cytokine production and positive regulation of tumour necrosis factor production (Figure S2C).

### 3.2 Definition of the IRGPI and validation its prognostic value for LGG patients

**FIGURE 4:**
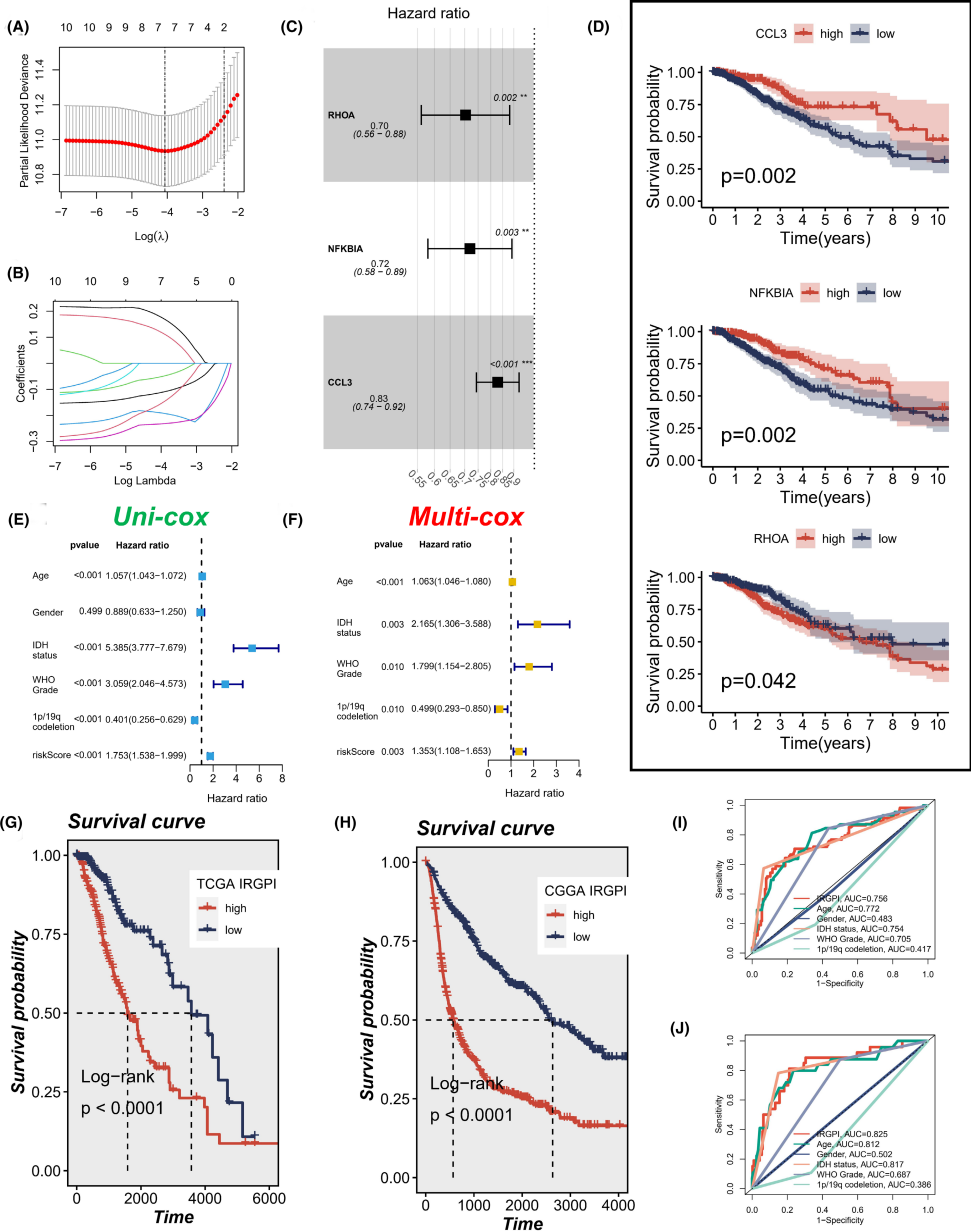
Definition of the IRGPI and survival analysis of different IRGPI subgroups for LGG patients. (A, B) Reduce the complexity of the model by applying LASSO regression analysis. (C). Univariate Cox analysis of 36 immune‐related hub genes and obtained three genes that significantly affect the OS of LGG patients to construct the prognostic index of all cancer samples. (D) The results with the Kaplan–Meier survival analysis for the three hub genes by the univariate Cox analysis. (E) Univariate Cox analysis was performed for clinicopathological factors and IRGPI score. (F) Multivariate Cox analysis was performed on the factors screened for significance in the univariate Cox analysis. (G) Kaplan–Meier survival analysis of the IRGPI‐high and IRGPI‐low subgroup in TCGA cohort. (H) Kaplan–Meier survival analysis of the IRGPI‐high and IRGPI‐low subgroup in CGGA cohort. (I) The predictive power of IRGPI signature in 1‐year (AUC = 0.756). (J) The predictive power of IRGPI signature in 3‐years (AUC = 0.825).

We further identified related genes to construct a prognostic index by using regression analysis. Initially, we selected 10 hub genes with *p* < 0.001 from the 36 identified immune‐related hub genes identified by univariate Cox regression analysis. In order to further narrow down the genes for constructing the model, we employed LASSO regression analysis to reduce the collinearity (Figure 4A,B), and ultimately identified three genes that significantly affect the OS of LGG patients, enabling us to construct a prognostic index for all cancer samples (Figure 4C,D). The formula of IRGPI was expressed as follows: IRGPI = (−0.352 × expression value of RHOA) + (−0.329 × expression value of NFKBIA) + (−0.187 × expression value of CCL3). Univariate Cox regression analysis showed that age, IDH status, WHO grade, 1p/19q codeletion and IRGPI were significantly associated with the prognosis of LGG (Figure 4E). Multivariate Cox regression analysis confirmed that IRGPI remained an independent prognostic factor for LGG after considering other clinical information (Figure 4F). The median IRGPI was used as the cut‐off value to divide LGG patients into two subgroups: IRGPI‐high and IRGPI‐low. In a survival analysis using TCGA cohort as a training set, we found that patients in IRGPI‐low subgroup had a better OS than those in IRGPI‐high subgroup (Figure 4G). Similarly, using CGGA cohort as the validation set, patients in IRGPI‐low subgroup also showed better outcomes than those in IRGPI‐high subgroup (Figure 4H). General baseline tables of patients in TCGA and CGGA cohort are presented in Tables S3 and S4. Moreover, we compared the ability of IRGPI to predict survival with other clinical factors, and the ROC curve results showed that our model had the optimal performance in 1‐year (Figure 4I) and 3‐years (Figure 4J).

### 3.3 Molecular mechanism of differential prognosis in two IRGPI subgroups

**FIGURE 5:**
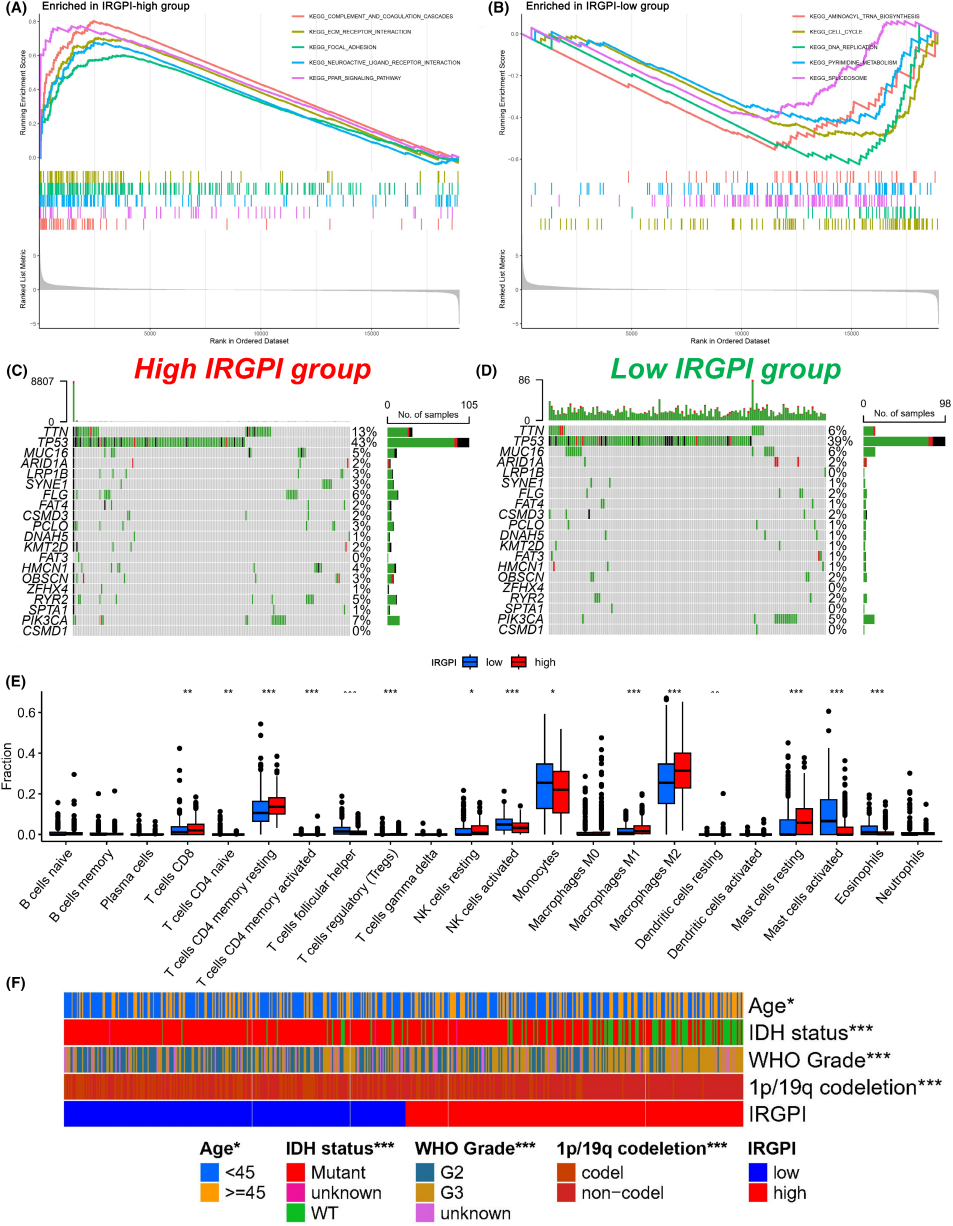
Molecular characteristics and immune landscape in the two IRGPI subgroups. (A) Gene sets enriched in IRGPI‐high subgroup. (B) Gene sets enriched in IRGPI‐low subgroup. (C, D) The major mutated genes in the mutated LGG samples of IRGPI‐high and IRGPI‐low subgroup. (E) The proportions of TME cells in different IRGPI subgroups. The scattered dots represent the immune scores of the two subgroups. The bold lines represent the median. The bottom and top of the boxes are 25% and 75%, respectively. Wilcoxon test was used for statistical analysis between the two subgroups (*p < 0.05, **p < 0.01, ***p < 0.001). (F) Age, IDH status, WHO Grade, 1p/19q codeletion and IRGPI were used as annotations for LGG patients.

We performed GSEA to identify gene sets‐enriched signalling pathways in different IRGPI subgroups. The gene sets of IRGPI‐high samples were primarily associated with ECM receptor interaction (Figure 5A), while gene sets enriched in IRGPI‐low samples were mainly related to DNA replication and cell cycle pathways (Figure 5B). Regarding gene mutation analysis, we observed a higher total mutation frequency in the IRGPI‐high subgroup compared to the IRGPI‐low subgroup, with missense mutation being the predominant mutation type in both groups, followed by nonsense mutation. Furthermore, we found that TTN, TP53, MUC16 and PIK3CA genes had mutation frequencies higher than 5% in both subgroups. Notably, TTN and TP53 mutations were the most common in both IRGPI‐high and IRGPI‐low subgroups (Figure 5C,D).

### 3.4 Immune mechanism of differential prognosis in two IRGPI subgroups

To investigate the immune mechanisms underlying differential prognosis in LGG patients, we compared the distribution of immune cells in different IRGPI subgroups using the Wilcoxon test. Our analysis revealed that activated NK cells, monocytes and eosinophils were more abundant in the IRGPI‐low subgroup, while regulatory T cells, resting NK cells and M2 macrophages were more abundant in the IRGPI‐high subgroup (Figure 5E). The results showed that the IRGPI‐low subgroup, which had a better prognosis, exhibited greater immune cell activation and more robust immune response. Figure 5F presents a detailed overview of the clinicopathological information and immunological landscape of the different IRGPI subgroups.

### 3.5 Relationship between IRGPI grouping and immunotherapy related indicators

**FIGURE 6:**
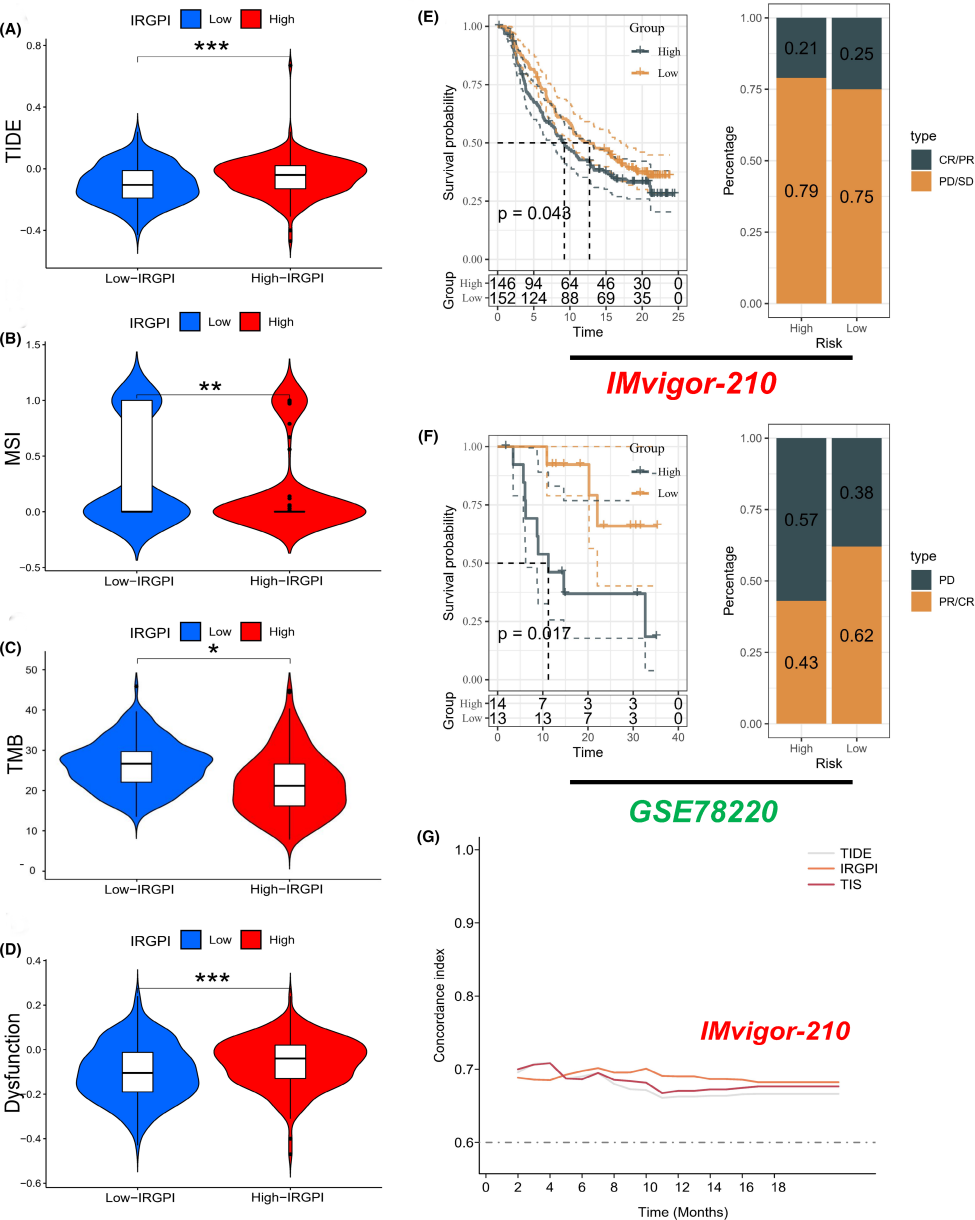
The prognostic value of IRGPI in patients after immunotherapy. (A–D) TIDE, MSI, TMB and T cell dysfunction score in different IRGPI subgroups (*p < 0.05, **p < 0.01, ***p < 0.001). (E) Kaplan–Meier survival analysis of the IRGPI subgroups in the cohort of IMvigor‐210 treated with anti‐PD‐L1 therapy. (F) Kaplan–Meier survival analysis of the IRGPI subgroups in the cohort of GSE78220 treated with anti‐PD‐1 therapy. (G) The Concordance‐index was used to compare the predictive power of IRGRI with TIS and TIDE in the cohort of IMvigor‐210. The C‐index of TIS and TIDE performed better in the first 5 months of follow‐up, while IRGPI performed better in predicting prognosis after 5 months of follow‐up.

We utilized multiple immunotherapy‐related indicators to evaluate the potential clinical efficacy of immunotherapy across different IRGPI subgroups. TIDE is a robust indicator that can effectively predict the response to immune checkpoint suppression therapy instead of a single biomarker. A higher TIDE score signifies a worse efficacy of ICIs, which may lead to no significant improvement in patients' survival after ICIs treatment. In our study, the TIDE score was higher in the IRGPI‐high subgroup than in the IRGPI‐low subgroup, indicating that patients in the latter subgroup were more likely to benefit from ICIs treatment than those in the former subgroup (Figure 6A). Furthermore, the IRGPI‐low subgroup had a higher microsatellite instability (MSI) (Figure 6B) and tumour mutational burden (TMB) score (Figure 6C), whereas the IRGPI‐high subgroup had a higher T cell dysfunction score (Figure 6D). These findings suggest that the IRGPI‐low subgroup was more responsive to immune drugs than the IRGPI‐high subgroup, indicating that patients were more likely to benefit from immunotherapy. Taking into account the results of previous prognostic analyses among different subgroups of IRGPI, we ultimately concluded that the IRGPI score had the potential to serve as a biomarker for clinically assessing the efficacy of immunotherapy in patients.

### 3.6 Validation of the prognostic value of IRGPI in patients after immunotherapy

To assess the prognostic value of IRGPI in patients receiving immunotherapy, we validated it by performing the prognostic analysis using the cohort of Imvigor‐210 (urothelial carcinoma, UC) treated with anti‐PD‐L1 therapy and GSE78220 (melanoma) treated with anti‐PD‐1 therapy. As shown in Figure 6E,F, we found that IRGPI‐low subgroup had a better prognosis than IRGPI‐high subgroup. This was consistent with our previous findings in both the TCGA and CGGA cohorts. In addition, we compared the predictive ability of IRGPI with the 18‐gene T‐cell‐inflamed signature (TIS) and TIDE in Mariathasan et al.'s UC cohort. Our study demonstrated that the C‐index of TIS and TIDE performed better in the first 5 months of follow‐up, while IRGPI performed better in predicting prognosis after 5 months of follow‐up (Figure 6G). Thus, we inferred that the prognostic value of IRGPI was comparable with that of TIS and TIDE for predicting survival in this UC cohort.

## 4 DISCUSSION

Glioma, a common malignant tumour in the CNS, is plagued by local invasive growth and excessive tumour heterogeneity.^18^ Although surgical resection, radiotherapy and chemotherapy are applied, the survival rate is still low.^19^, ^20^ In recent years, cancer immunotherapy has shown remarkable curative effects in many cancers.^21^, ^22^, ^23^ Immunotherapy strategies for glioma have been proposed and attracted extensive attention. However, a significant limitation of immunotherapy is that a large proportion of patients do not respond.^24^ Therefore, finding an effective biomarker to predict responsiveness to immunotherapy and improve overall response rates is an urgent research priority. In this study, we focused on developing a novel immunotherapy score to improve and aid the management of large‐sample‐based immunotherapy for patients with LGG.

WGCNA has been widely used in studying biological processes, providing a basis for discovering new immune‐related molecular biomarkers by constructing gene co‐expression networks and identifying modules to screen hub genes closely related to clinical phenotypes.^25^ In this study, we used WGCNA to identify 36 immune‐related hub genes that affect OS in LGG patients, and constructed IRGPI based on three identified genes, RHOA, NFKBIA and CCL3. In addition, multiple regression analysis showed that IRGPI could serve as an independent prognostic biomarker for immune‐related assessment of LGG patients, even after considering other clinical information. In the TCGA and CGGA cohorts, patients in the IRGPI‐low group had better OS than those in the IRGPI‐high subgroup.

We investigated the three genes comprising IRGPI to explore the possible mechanisms involved in IRGPI's role in immunotherapy. The ras homologue family member A (RHOA) gene encodes a member of the Rho family of small GTPases.^26^ When activated, it switches to an activated GTP‐bound form and promote actin cytoskeleton reorganization, which is associated with regulation of cell shape and multiple cellular functions.^27^, ^28^ RHOA has been demonstrated to be recurrently mutated in multiple types of cancer and played vital roles in the proliferation, migration and invasion of these tumour.^29^ RhoA has long been considered as an oncogene based on its mechanism of action by inducing RAS to transform into fibroblasts. Recent cancer genome‐wide sequencing studies have revealed the recurrence of loss‐of‐function RHOA hotspot mutations in primary leukaemia/lymphoma, suggesting a rather complex picture of RHOA function depending on cancer cell type.^30^ Besides, RHOA could modulate the TME. RHO‐kinase blockade increased cancer cell phagocytosis and enhanced of T cell priming, leading to suppression of tumour growth in syngeneic tumour models.^31^ Aberrant activation of nuclear factor kappa B (NF‐κB) is recognized as a crucial factor in cancer initiation and progression. The NF‐kappaB inhibitor alpha (NFKBIA) gene as a member of the family of NF‐κB inhibitors, it can regulate the NF‐κB into and out of the nucleus.^32^ The activity of NF‐κB in gliomas is significantly higher than that in normal tissues. In addition, NFKBIA was found to be eliminated in about 25% of grade IV gliomas. Reduced survival can be found in the NFKBIA deletion or low expression tumours. Correspondingly, when NFKBIA expression was restored in NFKBIA‐deficient tumour cells, the malignant phenotype was weakened and chemosensitivity was increased.^33^ Furthermore, NFKBIA correlates closely to the immune response. For instance, NFKBIA mRNA levels positively correlated with the infiltration levels of M1 macrophages, CD4^+^ T cells, CD8^+^ T cells and naive B cells in breast cancer.^34^ The C‐C motif chemokine ligand 3 (CCL3) is a chemokine that plays a key role in the formation of tumour immune microenvironment and tumour‐associated macrophages (TAMs) polarization. The change of chemokine expression level will affect the composition of tumour infiltrating immune cells and affect the tumour response to immunotherapy. Previous studies have shown that CCL3 can promote anti‐tumour immune response.^35^ Meanwhile, it has been implicated in tumour progression. However, some studies have shown CCL3 is overexpressed in a variety of tumour tissues and promotes tumours growth, and when applying an anti‐CCL3 neutralizing antibody, tumour growth reduction was observed.^36^ In summary, IRGPI is a biomarker associated with changes in the immune microenvironment and tumour suppression.

Gene mutations are frequent mechanisms leading to cancer. The type and frequency of mutations are closely linked to the prognosis of patients with cancer.^37^ In our study, we divided LGG patients into two subgroups based on their IRGPI scores. The gene mutation analysis revealed that the majority of the mutation types in these genes were amplifications, deep deletions and missense mutations. The TP53 mutation showed the largest difference between the IRGPI‐high and IRGPI‐low subgroups (43% vs. 39%), with a higher frequency in the former. The TP53 gene is the most well‐known tumour suppressor gene.^38^ TP53 mutations result in uncontrolled proliferation and invasive growth.^39^, ^40^ Furthermore, the IRGPI‐high subgroup displayed a higher mutation rate of PIK3CA than the IRGPI‐low subgroup (7% vs. 5%). Patients in the IRGPI‐high subgroup are likely to have a poorer prognosis due to a higher frequency of TP53 and PIK3CA mutations. Our results are consistent with the survival outcomes we observed.

We investigated the association between IRGPI and established predictive biomarkers for immunotherapy. TIDE was applied to evaluate the potential for tumour immune escape in the gene expression profile of tumour samples.^41^ Higher scores indicate poorer response to immunotherapy and shorter survival time. MSI is an established biomarker of immunotherapy. MSI‐H has been demonstrated to be an independent predictor of good prognosis in patients with stage II colorectal cancer.^42^ TMB refers to the total number of mutations per megabyte and a higher TMB leads to the production of more immune neoantigens. Moreover, the normal function of T cells plays a crucial role in tumour immunotherapy. We discovered that the IRGPI‐low subgroup exhibited lower TIDE scores, lower T cell dysfunction, and higher MSI and TMB, suggesting that the IRGPI‐low subgroup may have better responsiveness to immunotherapy. One of the mechanisms underlying this effect may be associated with lower levels of immune escape and fewer T‐cell defects. Additionally, the high mutation load caused by MSI makes the tumours more immunogenic, making patients relatively sensitive to immunotherapy. MSI is a common feature in LGG patients.^43^ To further validate the prognostic value of IRGPI, we performed the survival analysis on a UC cohort receiving anti‐PD‐L1 therapy and a melanoma cohort receiving anti‐PD‐1 therapy. We found that IRGPI can differentiate between different outcomes in patients treated with immunotherapy.

The TME plays important roles in regulating the immune response of cancer.^44^, ^45^ Understanding the status of TME and change the composition of TME can help to improve the response of LGG patients to immunotherapy. In this study, we observed notable differences in immune cell composition between the two IRGPI subgroups. We postulate that the relatively poor prognosis of patients in the IRGPI‐high subgroup may be attributed to a higher number of immunosuppressive cells in the TME or a lower immune response. Conversely, the TME of the IRGPI‐low subgroup demonstrated a more active immune response, with greater participation of effective immune cells.

As novel predictive biomarkers for immunotherapy, TIDE and TIS have shown greater accuracy in predicting the response of patients receiving immunotherapy.^41^, ^46^ TIDE was developed as a creative computational method to identify factors underlying mechanisms of tumour immune escape: induction of T cells dysfunction in tumours with high CTL infiltration and prevention of T cells infiltration with low CTL levels. TIS was designed to better reflect the degree of immune cell infiltration in the TME. Based on the interferon signalling pathway, the researchers further added eight other genes related to the infiltration and abundance of immune cells in the TME.^47^ However, these methods have limitations, as they primarily focus on T cell function and do not fully capture the complexity of the TME involvement in immunotherapy response.^22^ Additionally, they are more concerned with patients' response to immunotherapy rather than their OS time. By contrast, our study found that the predictive value of IRGPI for OS was comparable to that of TIDE and TIS, but differed over time. While TIDE and TIS performed better during the 5‐month follow‐up period, the predictive power of IRGPI was superior after the 5‐month follow‐up. Therefore, IRGPI may be a better predictor of OS over longer follow‐up periods. Moreover, IRGPI only involves three genes, making it convenient to detect. Our findings suggest that IRGPI could serve as a valuable tool for predicting the prognosis of LGG patients receiving immunotherapy.

IRGPI may has a wide application prospect for LGG patients in future. The first is the screening of immunosensitive patients. In recent years, tumour immunotherapy has shown remarkable curative effects in many cancers.^21^, ^22^, ^23^ However, there are obstacles to the promotion of immunotherapy strategies for glioma.^24^ In our study, we investigated the three genes comprising IRGPI as a biomarker associated with prognosis. Genetic analysis of patients can be performed to see how these three genes are expressed in the body to help clinicians develop immunotherapy strategies. In addition, we found that the potential mechanism for the differential prognosis between the two IRGPI subgroups may be related to the frequency of genetic mutations in LGG patients, such as patients in the IRGPI‐high subgroup have a higher frequency of TP53 and PIK3CA mutations. The other aspect is related to the change of the TME. That is, the tumours may change the composition of immune cells in its microenvironment. Therefore, patients with high IRGPI scores can use some drugs targeting TP53 and PIK3CA, or take some immune cell activation to improve the long‐term prognosis of patients. Finally, the advantage of IRGPI in predicting the prognosis of patients receiving immunotherapy. IRGPI could play an important role in clinical decision‐making, guiding treatment strategies and evaluating prognosis.

There were some limitations to our study. The sample size and retrospective approach limited the study's ability to draw causal conclusions. We tried to analyse potential confounders that could affect the prognostic value of IRGPI as comprehensively as possible, but there were still individualization of patients and heterogeneity of treatment methods. In addition, the relationship between IRGPI and TME was initially explored in our study. However, the mechanisms that the activation and recruitment of immune cells needs to be further validated in prospective studies with larger patients' cohorts.

## 5 CONCLUSION

In conclusion, the grouping of IRGPI serves as a valuable biomarker for assessing the response and efficacy of immunotherapy in LGG patients. Our study revealed that IRGPI not only distinguishes patients based on their molecular and immunological characteristics but also predicts their prognosis more accurately. As a potential prognostic indicator of immunotherapy, IRGPI holds significant promise for clinical application.

## AUTHOR CONTRIBUTIONS

**Jing Zhou:** Data curation (equal); formal analysis (equal); methodology (equal); software (equal); writing – original draft (equal). **Hao Guo:** Conceptualization (equal); project administration (equal). **Likun Liu:** Data curation (equal); investigation (equal); methodology (equal); software (equal). **Zengcai Jin:** Investigation (equal); software (equal); validation (equal). **Wencui Zhang:** Software (equal); validation (equal). **Tao Tang:** Conceptualization (equal); project administration (equal); resources (equal).

## CONFLICT OF INTEREST STATEMENT

The authors declare that there is no conflict of interest.

## Supporting information


Figure S1.
Figure S2.

Table S1.

Table S2.

Table S3.

Table S4.

## Data Availability

The data generated or analysed of this study are derived from the TCGA database, CGGA database and GTEx database, which are publicly available databases.
