# Supplementary material for: Identification of immune‐related hub genes and construction of an immune‐related gene prognostic index for low‐grade glioma
Source: J Cell Mol Med. 2023 Sep 29;27(23):3851–63. doi: 10.1111/jcmm.17960 (PMC10718158; doi:10.1111/jcmm.17960)
Supplement: Supplementary file 1 — Figure S1. Figure S2. [file JCMM-27-3851-s003.docx]

**IDENTIFICATION OF IMMUNE-RELATED HUB GENES AND CONSTRUCTION OF AN IMMUNE-RELATED GENE PROGNOSTIC INDEX FOR LOW-GRADE GLIOMA**

SUPPLEMENTARY FIGURES


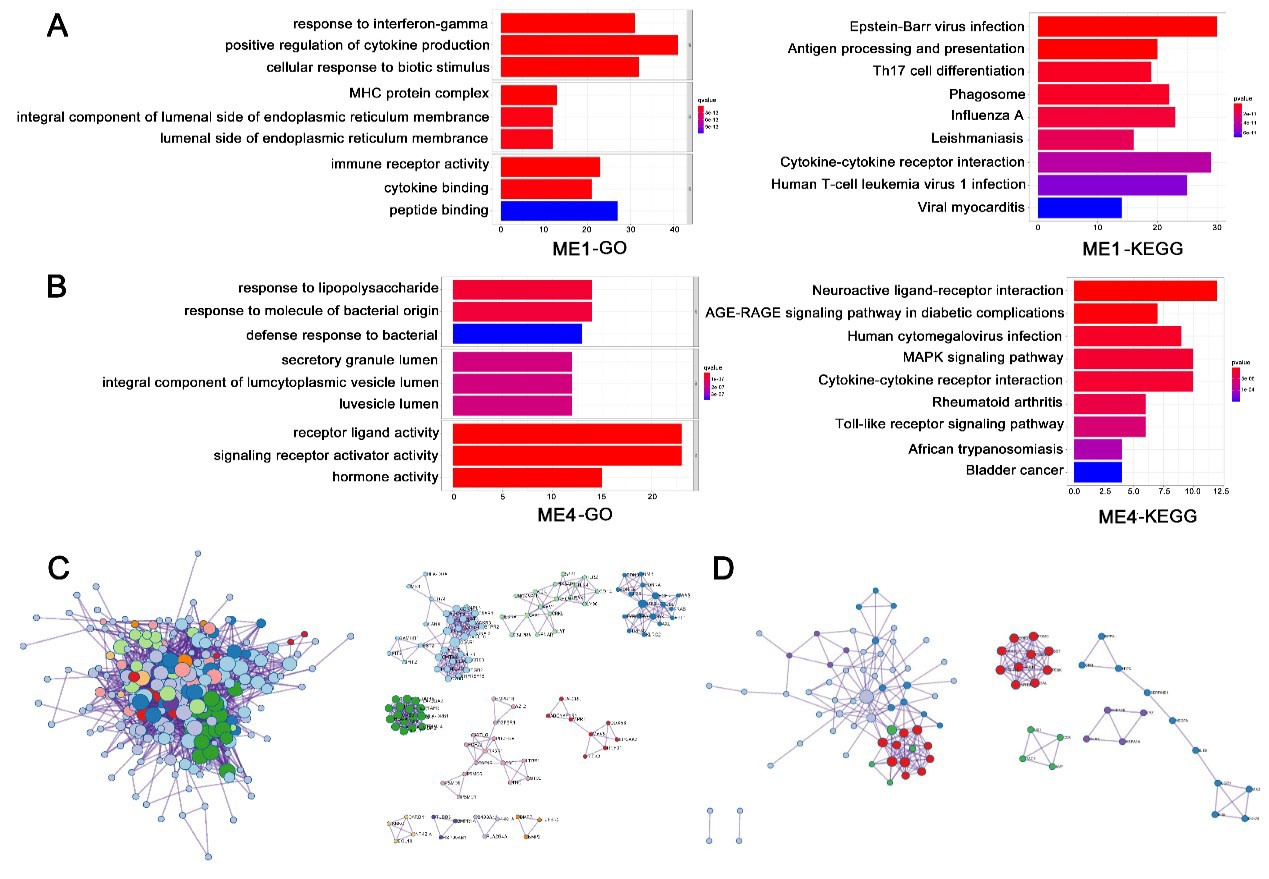


**Figure S1. GO and KEGG pathway enrichment analysis of the modules by WGCNA and identification of immune-related hub genes.**

**(A)** Gene Ontology (GO) and Kyoto Encyclopedia of Genes and Genomes (KEGG) pathways enriched in the genes of the ME1 module. **(B)** GO and KEGG pathways enriched in the genes of the ME4 module. **(C)** The network of the genes in the ME1 module. **(D)** The network of the genes in the ME4 module.


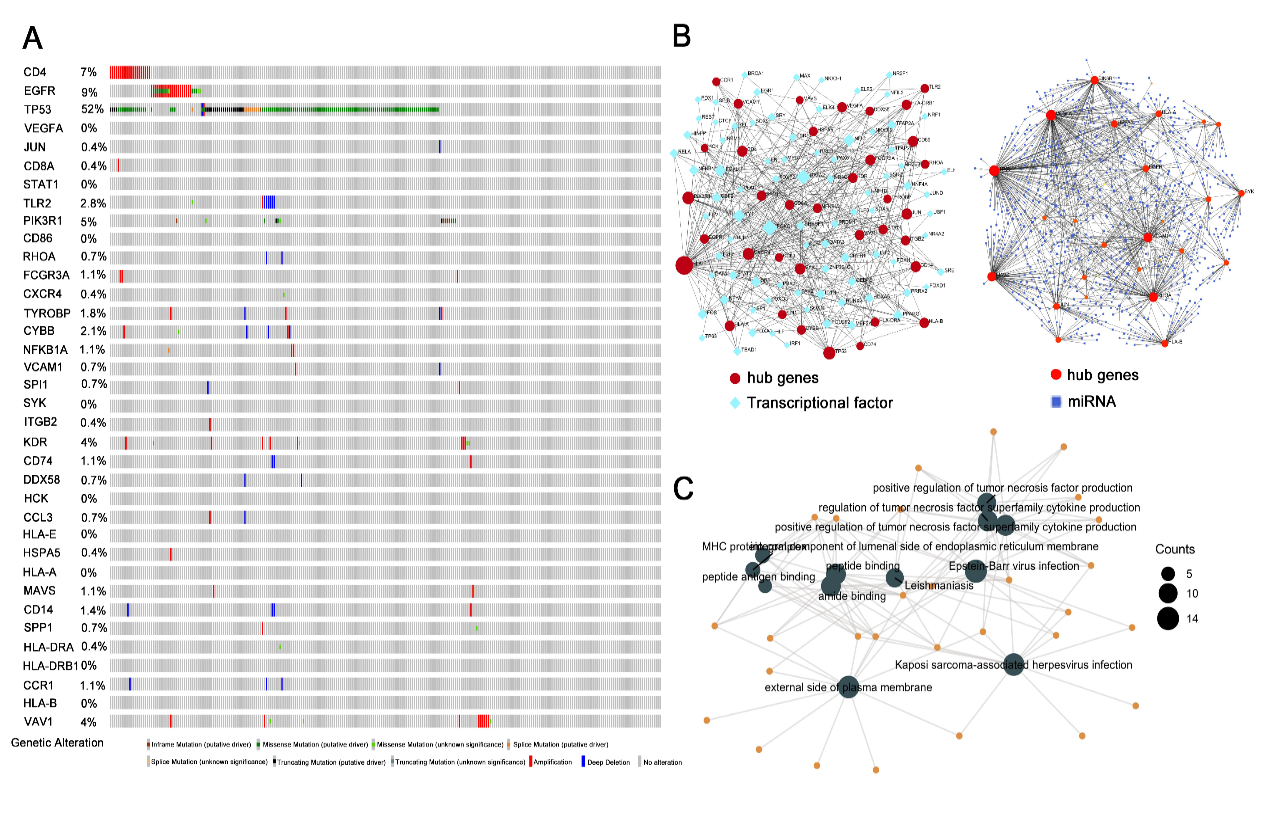


**Figure S2. Molecular characteristics of immune-related hub genes.**

**(A)** The results of gene mutation analysis for 36 immune-related hub genes are displayed. The mutation frequency of TP53, EGFR, CD4 and PIK3R1 were more than 5%. **(B)** The network summarizes complex connections among immune-related hub genes, transcription factors and miRNA. The size of the node is positively correlated with the degree of the node. **(C)** The KEGG pathway enrichment analysis of immune-related hub genes are shown in the regulatory network. The size of the circle

indicates the number of genes in the enrichment pathway.
